# Timely stereotactic body radiotherapy (SBRT) for spine metastases using a rapidly deployable automated planning algorithm

**DOI:** 10.1186/s40064-016-2961-3

**Published:** 2016-08-11

**Authors:** Omar Y. Mian, Owen Thomas, Joy J. Y. Lee, Yi Le, Todd McNutt, Michael Lim, Daniele Rigamonti, Jean-Paul Wolinsky, Daniel M. Sciubba, Ziya L. Gokaslan, Kristin Redmond, Lawrence Kleinberg

**Affiliations:** 1 Department of Radiation Oncology, Cleveland Clinic, Taussig Cancer Institute, Cleveland, OH USA; 2 Delmarva Radiation Services, Tunnell Cancer Center, Rehoboth Beach, DE USA; 3 Department of Radiation Oncology and Molecular Radiation Sciences, Sidney Kimmel Comprehensive Cancer Center, The Johns Hopkins University School of Medicine, Baltimore, MD USA; 4 Department of Radiation Oncology, Indiana University, Indianapolis, IN USA; 5 Department of Neurosurgery, The Johns Hopkins University School of Medicine, Baltimore, MD USA

**Keywords:** Automated planning, SBRT, Radiation therapy, Spinal lesions

## Abstract

**Purpose/objectives:**

The complex planning and quality assurance required for spine SBRT are a barrier to implementation in time-sensitive or limited resource clinical situations. We developed and validated an automated inverse planning algorithm designed to streamline planning and allow rapid delivery of conformal single fraction spine SBRT using widely available technology.

**Materials/methods:**

The Rapid Spine (RaSp) automated script successfully generated single fraction SBRT plans for fourteen complex spinal lesions previously treated at a single high-volume institution. Automated RaSp plans were limited to 5 beams with a total of 15 segments (allowing calculation-based verification) and optimized based on RTOG 0631 objectives. Standard single fraction (16 Gy) stereotactic IMRT plans were generated for the same set of complex spinal lesions and used for comparison. A conservative 2 mm posterior isocenter shift was used to simulate minor set-up error.

**Results:**

Automated plans were generated in under 5 min from target definition and had a mean dose to the PTV of 1663 cGy (SD 131.5), a dose to 90 % of PTV (D90) of 1358 cGy (SD 111.0), and a maximum point dose (Dmax) to the PTV of 2055 cGy (SD 195.2) on average. IMRT plans took longer to generate but yielded more favorable dose escalation with a mean dose to the PTV of 1891 cGy (SD 117.6), D90 of 1731 cGy (SD 126.5), and Dmax of 2218 cGy (SD 195.7). A 2 mm posterior shift resulted in a 20 % (SD 10.5 %) increase in cord dose for IMRT plans and a 10 % (SD 5.3 %) increase for RaSp plans. The 2 mm perturbation caused 3 cord dose violations for the IMRT plans and 1 violation for corresponding RaSp plans.

**Conclusion:**

The Rapid Spine plan method yields timely and dosimetrically reasonable SBRT plans which meet RTOG 0631 objectives and are suitable for rapid yet robust pretreatment quality assurance followed by expedited treatment delivery. RaSp plans reduce the tradeoff between rapid treatment and optimal dosimetry in urgent cases and limited resource situations.

## Background

Spine metastases are a common complication of malignancy, leading to diminished performance status, pain, and neurologic compromise. Radiotherapy is the standard for non-invasive management of spine metastases in patients without mechanical instability or symptomatic spinal cord compression. Single fraction conventional treatments such as 800 cGy have been shown to be equivalent to fractionated treatment (e.g., 3000 cGy in 10 fractions) in providing pain relief from metastatic disease of the spine without dose limiting or late complications such as myelopathy (Hartsell et al. 2005; Howell et al. 2013). Nevertheless, as many as 60 % of patients treated with conventional spine radiotherapy respond inadequately to treatment and retreatment is common. Increasingly conformal radiotherapy methods employing stereotactic set-up have allowed precise and safe delivery of higher doses of radiation to the tumor volume with durable local control rates approaching 90 % (Gerszten et al. 2007). A growing body of clinical data suggests stereotactic body radiation therapy (SBRT) is well tolerated and provides rapid and durable pain relief (Ryu et al. 2011; Amdur et al. 2009; Schipani et al. 2012). SBRT has the added benefit of shortened overall treatment course and smaller treatment volumes with less radiation exposure to adjacent normal tissues, potentially maximizing access to the systemic therapies important in this group of patients. Recent prospective trials including an ongoing cooperative group study (RTOG 0631) are further investigating whether clinical outcome measures such as pain control are improved with stereotactic radiotherapy compared to conventional radiation therapy.

In general, intensity modulated radiotherapy (IMRT) involves inverse planning based on user-defined dosimetric constraints and relies on more complex and dynamic segmentation of beams compared to other conformal techniques, such as forward planned 3D conformal radiotherapy. While other methods utilize multi-leaf collimator beam shaping and can employ stereotactic immobilization, IMRT plans are generally thought to produce more conformal therapeutic volumes by iterative, computation-intensive inverse planning. There are, however, several practical disadvantages to the use of IMRT based stereotactic treatments for spine metastases. In resource limited situations, the availability of trained dosimetrists, planning systems, and linear accelerators equipped to perform IMRT based stereotactic therapy are limited. When available, the complex inverse planning and requirement for IMRT patient-specific quality assurance (QA) can delay the start of treatment and limit the utility of IMRT in the urgent setting. In the appropriate scenario, calculation-based verification is sufficient and is considered both standard and robust (Lo et al. 2013). In addition, the steep dose gradients with highly conformal treatments require labor-intensive stereotactic treatment set-up and verification which can lengthen the treatment time, limit compliance and feasibility in patients with severe tumor-related pain, and increase cost and resource requirements. With these limitations to IMRT in mind, alternative strategies for the rapid and safe delivery of conformal single fraction radiotherapy are desirable. Although planning systems designed to speed the process have recently become commercially available, such resources are not widely available in the United States and worldwide. The present work explores the use of an automated inverse planning workflow (Rapid Spine) designed to minimize planning time, generate rapid high quality plans with limited beam segmentation, and utilize non-IMRT calculation-based verification while adhering to RTOG spine SBRT guidelines and dosimetric constraints.

## Methods

### Patient selection and target lesions

Fourteen spinal lesions previously treated at a high volume institution with either conventional or stereotactic radiation therapy had new single fraction treatment plans automatically generated using the Pinnacle Planning System and automating scripts. The scripts used in planning are available from the corresponding author. All patients were simulated using a non-contrast enhanced CT scan in the supine position with standard immobilization techniques. Twelve lesions abutted the thecal sac, eight involved spinal pedicles, seven demonstrated epidural extension, and two were circumferential. It is worth noting that controversy exists as to the optimal management in cases of epidural extension in light of data supporting downstaging with minimally invasive separation surgery prior to SBRT (Laufer et al. 2013). In the present study, three patients had undergone debulking surgery, one patient underwent pretreatment vertebroplasty, and the remainder were not considered candidates for surgical decompression. All patients included had disease that was considered clinically urgent. Eight tumors were thoracic, five were lumbar, and one was cervical. Details including histology, spinal column level, planning target volume (PTV) volume, associated symptoms, and lesion characteristics are given in Table 1.

**Table 1:** Histology and lesion characteristics

ID	Diagnosis	Level	Lesion characteristics	Circumferential	Symptoms	Volume CTV (cc)
1	Spindle cell	T8	Epidural extension, compression, T2 Cord changes	No	Pain, Non focal	29.192
2	Esophageal Adeno	L3	Wedge deformity, no canal compromise	No	Pain, Non focal	41.86
3	Chordoma	L2	Pedicle involvement, Mass effect on thecal sac	Pedicle	Pain, Non focal	82.36
4	Adrenal	L3	Circumferential PTV, debulking of L3, vertebrectomy	Yes	Pain, Non focal	182.8
5	Prostate	L5	Retropulsion with canal narrowing, pedicle involvement	Pedicle	Pain, urostomy	68.14
6	Breast	T12	Expansile s/p vertebroplasty, pedicle but no canal	Pedicle	Pain	46.16
7	Pancreatic	T8-9	Circumferential, T9 compression, epidural extension	Yes	Pain	129.932
8	Glottic SCC	T2	s/p Laminectomy from T2–T4	No	Pain, ataxia	5.87
9	Melanoma	L5	b/l Pedicles with epidural extension	Pedicle	Pain, proprioception	37.75
10	Renal cell	T8	s/p Resection	Pedicle	Pain	137.61
11	Colon	T12	Extension into central canal with cord displacement	Pedicle	Pain	63.57
12	Renal cell	T8	Posterior pedicle involvement and flattening of cord	Pedicle	Pain	26.36
13	Renal cell	C7	No pedicle, no epidural extension	No	Pain	16.84
14	Renal cell	T3	Pedicle involvement	Pedicle	Pain	27.06

### Rapid Spine (RaSp) script

**Fig. 1:**
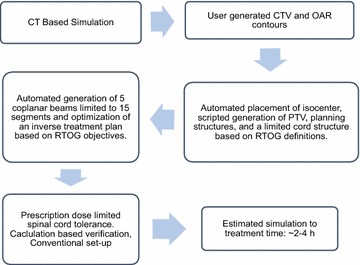
RaSp workflow

An automated inverse planning script was developed using the Pinnacle Planning System with the goal of yielding a robust algorithm producing systematized treatment plans applicable to varied and complex cases with minimal need for user individualization. Parameters for the script included the delivery of stereotactic doses to the PTV up to the RTOG 0631 standard of 16 Gy in a single fraction. The spinal cord was contoured starting 10 cm above the superior extent of the PTV to 10 cm below the inferior extent of the PTV. If necessary, the algorithm limited the prescription dose to meet spinal cord constraints as described further below. The clinical target volume (CTV) was manually defined by the treating physician according to international consortium guidelines (Cox et al. 2012). For a given plan, an automated script automatically placed the isocenter at the calculated volumetric centroid point of the target volume, generated a PTV (2 mm uniform expansion of the CTV, excluding the thecal sac), concentric ring ROIs for planning, and a truncated thecal sac (limited to the superior/inferior dimensions of the PTV). A second sequential script placed either 2, 3, 5, or 7 coplanar beams, which were limited to 3 or fewer segments per beam. The same script then optimized the treatment plan in inverse fashion based on fixed treatment objectives following RTOG 0631 protocol for dose specifications, target coverage, cord dose limits (Fig. 1). For each patient, the final plan was optimized: first, a plan was generated using a conservative objective of a point dose maximum to the thecal sac of 12 Gy; if this plan did not meet cord constraints, then an alternate plan using an objective of 10 % of the truncated thecal sac receiving less than 10 Gy was generated. Both plans were evaluated for adherence to treatment objectives. If the objectives on the thecal sac were not met by inverse planning, the prescription dose was lowered to meet these objectives. A hard constraint was placed on cord dose so as not to exceed a point maximum of 12 Gy with a dose to 10 % of a limited thecal sac structure (as defined above) of less than 10 Gy.

### *In silico* simulation of setup error

A 2 mm anterior/posterior shift was manually introduced by moving the isocenter posteriorly toward the spinal cord to simulate a ‘worst case scenario’ set-up error. A 2 mm error was chosen to represent the realistic possibility that set-up errors of this small magnitude may routinely occur and go undetected, particularly when standard, non-stereotactic immobilization is used—a likelihood that is increased in cases where urgent therapy is required. Dosimetric endpoints including CTV coverage and cord dose were obtained before and after this shift and compared to determine the impact of set-up error on both IMRT as well as scripted RaSp treatment plans. The intended treatment linear accelerator employed a multi-leaf collimator (MLC) leaf size between 5 and 10 mm.

## Results

**Fig. 2:**
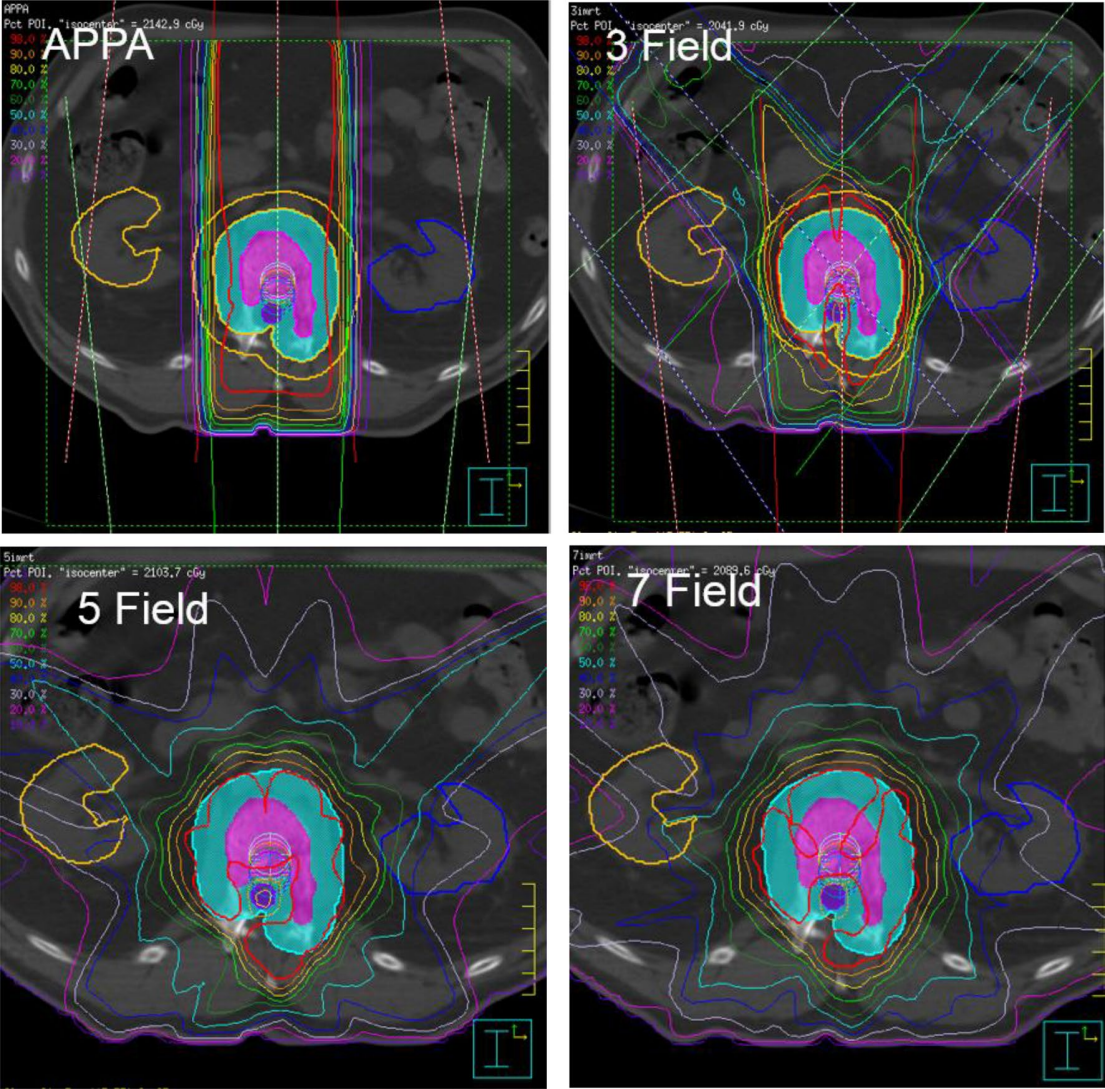
Representative plan images comparing 2-, 3-, 5-, and 7-field RaSp plans

Fourteen spinal lesions were examined with a mean CTV volume of 63.97 cc (range 5.87–182.8). The CTV included a single vertebral level for all but one patient, where two vertebral levels were included. Histology and lesion characteristics varied (Table 1). Single fraction IMRT (16 Gy) and 2-, 3-, 5-, and 7-field RaSp automated plans were generated for each lesion to cover the target volume as described in the “Methods” section. For automated RaSp plans, increasing beam number resulted in more conformal automated plans: the 5-field plans qualitatively yielded the best balance between coverage and plan simplicity and robustness (Fig. 2). 5-field plans, limited to 3 segments or fewer per beam, were used for subsequent comparisons with corresponding IMRT plans. All automated plans were generated and optimized in under 5 min. In comparison, IMRT plans took longer to generate, as planning varied from 2 to 4 h. For complex lesions, IMRT plans often took more than 4 h to complete multiple iterations during plan optimization. In addition, IMRT plans had a higher average beam number, and higher total segment count (7.1 [SD 1.0] beams and 52.6 [SD 11.7] segments).

**Fig. 3:**
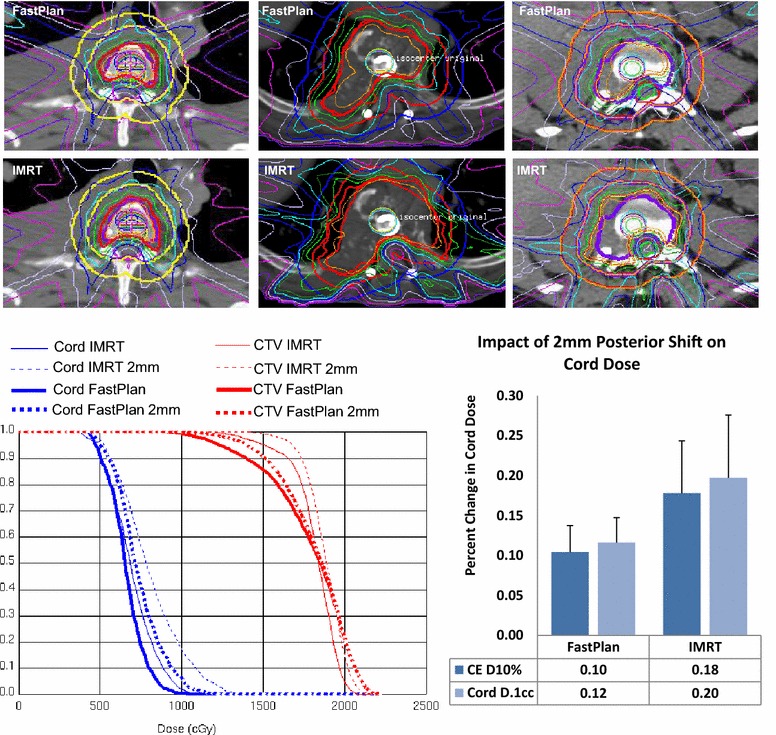
Plan comparison. *Top panel* Representative 5-field RaSp and IMRT plans with a 2 mm isocenter shift to simulate setup error. *Bottom Left* Dose-volume histogram plots of representative cord and CTV dose for both IMRT and RaSp based plans with and without the 2 mm shift.  *Bottom Right* *Bar graph *shows the percent change in cord dose (Cord Eval max 10 % and Cord Max to 0.1 cc) following the 2 mm AP shift. *Bars *represent mean percent change for all 14 lesions; *error bars *show 95 % CI

RaSp plans yielded a mean dose to the PTV of 1663 cGy (SD 131.5, 95 % CI 1582.2–1745.1), dose to 90 % of the PTV (D90) of 1358 cGy (SD 111.0, 95 % CI 1289.2–1426.8), and a maximum point dose (Dmax) to the PTV of 2055 cGy (SD 195.2, 95 % CI 1934.2–2176.3), on average. IMRT plans yielded more favorable dose escalation with a mean dose to the PTV of 1891 cGy (SD 117.6, 95 % CI 1818.3–1964.1), D90 of 1731 cGy (SD 126.5, 95 % CI 1652.5–1809.5), and Dmax of 2218 cGy (SD 195.7, 95 % CI 2097.1–2339.7) (Fig. 3). Mean point minimum doses were lower but not significantly different between RaSp (846.7 cGy, 95 % CI 715.3–978.1) and IMRT plans (1035.7 cGy, 95 % CI 875.2–1196.3).

All initial RaSp and IMRT plans met RTOG 0631 spinal cord limits. As stipulated in 0631, the spinal cord dose remained below 10 Gy to 10 % of the partial spinal cord volume defined as 5–6 mm above and below the target. In no case was the dose of 10 Gy exceeded for a spinal cord volume of 0.35 cc. The absolute maximum dose to the spinal cord remained below 14 Gy for a volume of 0.03 cc. Any spinal cord dose that did not meet these criteria would have been considered a major deviation in RTOG 0631 and was therefore considered unacceptable for either IMRT or RaSp plans in this study. Treatment plans were considered adequate as long as 90 % of the target volume received the prescribed radiosurgery dose and, as in RTOG 0613, dose inhomogeneity within the target volume was allowed, provided high dose spillage was limited to 105 % of the prescription dose outside of the PTV volume.

We compared plan conformality using an R100 % (conformality index) as well as intermediate-dose spillage determined from D2 cm and R50 % parameters. As expected IMRT plans demonstrated a trend towards increased conformality and steeper dose gradients when compared to RaSp plans, with lower R100 % as well as lower D 2 cm, and R50 % respectively. These differences, however, were not statistically significant, perhaps due to insufficient power given the number of lesions in the present analysis as well as the wide variation in PTV volumes. R50 % and D 2 cm parameters in particular are vulnerable to volume variations and guidelines typically account for this with target volume specific cut offs (e.g., RTOG 0915 lung SBRT guidelines).

**Fig. 4:**
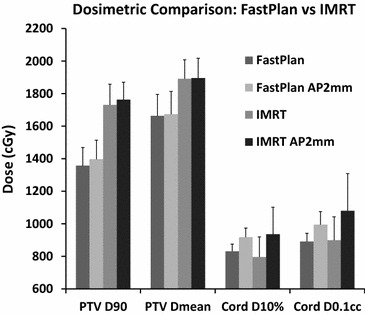
RaSp vs IMRT. Average dose to 90 % of the PTV (PTV D90), mean dose to PTV (PTV Dmean), maximum dose to 10 % of the cord (Cord D10 %), and maximum cord dose to 0.1 cc of the cord structure (Cord D0.1 cc) are plotted for IMRT and RaSp with and without a 2 mm AP shift to simulate set-up error. IMRT plans yielded more favorable dose escalation with a higher average PTV dose. A 2 mm shift had a greater relative impact on maximum cord dose for IMRT plans compared to RaSp plans

A 2 mm posterior shift (high dose volume shifted towards the cord), was introduced in silico to simulate a small amplitude, worst case set-up error. The 2 mm shift resulted in a 20 % (SD 10.5 %) increase in cord dose for IMRT plans and a 10 % (SD 5.3 %) increase for RaSp plans on average due to the steeper dose gradient with IMRT (Fig. 4). The same 2 mm anterior/posterior perturbation caused 3 cord dose violations for the IMRT plans and 1 violation for corresponding RaSp plans, illustrating a potential vulnerability of increasingly dose escalated conformal plans with steep dose fall off.

## Discussion

The spine is the most frequent site of skeletal metastatic disease, accounting for nearly 20,000 new cases per year in the United States (Sciubba et al. 2009). Radiotherapy remains a mainstay of treatment for spine metastases and there is mounting evidence for the utility of stereotactic radiotherapy in preoperative as well as postoperative settings (Kaloostian et al. 2014; Chawla et al. 2009; Sahgal et al. 2011). However, limitations in SBRT as described previously have lead us to develop an alternative planning process. We have explored the feasibility and utility of an automated script for the rapid generation of high quality conformal spine radiotherapy plans that could be planned and delivered with widely available resources. Here we report the preclinical validation of the “Rapid Spine” algorithm, an easily deployable and robust series of Pinnacle scripts which automate the creation of timely, dosimetrically reasonable radiotherapy plans designed to meet cooperative group standards for spine SBRT. The clinical need for such a strategy can be distilled down to two basic assumptions: (1) conformal stereotactic treatment is preferable to conventional single fraction radiotherapy for spine metastases and (2) the ‘standard’ IMRT workflow for stereotactic spine treatment impedes timely treatment in situations where dose escalation may otherwise be desirable.

Prospective studies have demonstrated equivalence of single fraction radiotherapy relative to fractionated regimens for pain control (Nielsen et al. 1998; Kaasa et al. 2006). Now mounting data from multiple studies have demonstrated excellent local control and durable pain relief with dose escalation above 8 Gy. Importantly, these studies have demonstrated efficacy without compromising safety, with low rates of complications such as radiation-induced myelopathy. In 2008, Ryu et al. reported on 61 previously unirradiated solitary spine metastasis treated at the Henry Ford Hospital to doses between 10 and 16 Gy, delivered to the vertebral body and paraspinal soft tissue (Ryu et al. 2008). In this study, the median duration of pain relief was 13.6 months. One year overall survival was 74 % and no cord injury was observed. Similarly, at the University of Pittsburgh, Gerszten et al. treated 393 patients with 500 spinal metastases of varying histology and including 344 previously irradiated lesions (Gerszten et al. 2007). The dose range was 12.5–25 Gy and again, no cases of radiation-induced myelopathy were reported. Long-term pain improvement was noted in 86 % of patients at a median follow up of 21 months with a radiographic control rate of 75 % for melanoma, 87 % for renal cell carcinoma, and 100 % with breast and lung carcinomas. Similar outcomes were reported by Yamada et al. at Memorial Sloan-Kettering Cancer Center (103 lesions, 93 patients, doses 18–24 Gy, no myelopathy) and Gibbs et al. at Stanford (102 lesions, 74 patients, 16–26 Gy, 3 cases of myelopathy—2 of which were in previously irradiated patients) (Yamada et al. 2008; Gibbs 2007).

Despite these and other provocative data intimating the potential superiority of stereotactic radiotherapy, few generally accepted principles exist as to the criteria for routine management of spine metastases with dose escalated radiotherapy (Hall et al. 2011; Thibault et al. 2014). The NRG has taken up this questions and the ongoing study 0631 seeks to prospectively assess improved clinical outcomes with spine SBRT using single fraction stereotactic treatment (16 Gy). The recently published phase 2 results from this study demonstrate that SRS is feasible without any grade 4/5 treatment related toxicity for the initial 46 patients (Ryu et al. 2014).

In light of emerging data and ongoing studies, stereotactic radiotherapy has gained increasing acceptance as a primary modality in the routine management of spine metastasis. However, in its present form, IMRT-based stereotactic radiotherapy with stereotactic setup imposes logistic barriers to timely treatment in urgent and limited resource settings. Certainly, rigorous standards for treatment planning, quality assurance, and institutional credentialing are appropriate given the potential risks of SBRT (Gerszten et al. 2013). More efficient strategies are nevertheless worth exploring, as they may increase access to include patients who may benefit from dose escalation in time-sensitive cases or at institutions with limited access to resources.

The present work reports on a pilot study validating a novel approach aimed at improving efficiency through the utilization of automated treatment planning scripts. The integrated RaSp workflow consolidates the steps involved in stereotactic treatment planning, including CT based simulation, contouring, inverse treatment planning with limited beam segmentation, and finally, standard calculation based verification (Fig. 1). The RaSp treatment planning algorithm is customizable and relies at its core on a risk adapted approach, limiting the final prescription by dose to the spinal cord. The simulation parameters as well as physician contouring and delineation of target and OAR structures are identical for both RaSp and standard approaches.

Automated RaSp plans were generated quickly (on the order of minutes) after target and OAR structures were defined and yielded very reasonable dosimetry including a mean dose to the PTV of 1663 cGy after limiting dose to the spinal cord to RTOG 0631 limits. As expected, RaSp plans were less conformal than corresponding IMRT plans as reflected in a trend towards higher R100 %. The increased beam number and segmentation utilized for IMRT plans (necessitating more labor-intensive IMRT QA for verification) resulted in a steeper dose gradient at the PTV/Cord interface, as well as a trend towards lower D 2 cm and R50 % intermediate dose spillage, allowing more favorable dose escalation with a mean dose to the PTV of 1891 cGy—13 % higher than corresponding RaSp plans on average. Target coverage was similarly improved with IMRT with a 22 % increase in PTV D90 over corresponding RaSp plans, on average.

The steeper dose gradient observed with IMRT led us to hypothesize that IMRT-based plans may be more sensitive to setup error. Indeed we found that a 2 mm anterior/posterior shift in the isocenter resulted in a 20 % (SD 10.5 %) increase in cord dose for IMRT plans and a 10 % (SD 5.3 %) increase for RaSp plans on average. The same 2 mm perturbation caused 3 cord dose violations for IMRT plans and only 1 violation for corresponding RaSp plans. The smaller dosimetric impact of setup error suggests that appropriate safety may be maintained even during urgent therapy of patients in distress. Furthermore, in urgent situations where surgery may be preferable to SBRT, for example in cases of extensive epidural extension of disease (Patchell et al. 2005; Sahgal et al. 2008; Laufer et al. 2013), RaSp may offer an alternative approach for safe dose escalation in persons otherwise ineligible for surgical decompression. While further investigation is required to improve the process proposed here, the prospect of providing a safer and faster option for dose escalation radiation treatment to a broader spectrum of patients nationally and worldwide is intriguing.

On the whole, the RaSp algorithm efficiently and robustly yielded dosimetrically reasonable plans when compared to more resource-intensive IMRT-based spine radiotherapy. When feasible, more complex planning (e.g. IMRT, CyberKnife Multiplan) is preferable given its increased conformality and the possibility of increased dose escalation. However, in urgent situations, rapid initiation of treatment is particularly important, especially where invasive decompression is contraindicated. Speed of treatment initiation may be of importance in cases of symptomatic and asymptomatic cord compression, and thus may be a value to balance against the potential benefit of further dosimetric optimization. Also, in limited resource situations where SBRT may be unavailable, patients may benefit from a safe, time efficient, and robust method for planning dose escalated single fraction radiotherapy.

Implementations strategies for the clinical utilization of RaSp scripting are in development with a focus on optimizing more complex beam geometries yielding more favorable conformality, publishing open-source scripts for multiple treatment planning platforms, and prospective validation in early phase prospective clinical trials. With the use of automated scripts, the marginal reduction in conformality must be weighed against the benefit of expeditious treatment. Such tradeoffs are appropriately made in other clinical circumstances varying from urgent radiation techniques to emergent invasive surgeries, and prospective clinical validation with RaSp is the necessary next step in setting a standard for the ideal balance between time and conformality. The Rapid Spine approach reduces the tradeoff between rapid treatment and optimal dosimetry in urgent situations, warranting further development and clinical validation of this approach.

## Abbreviations

cGy: centigray
CTV: clinical target volume
Dmax: maximum point dose
Gy: gray
IMRT: intensity-modulated radiation therapy
PTV: planning target volume
QA: quality assurance
RaSp: Rapid Spine
SBRT: stereotactic body radiation therapy
SD: standard deviation
